# A Case of Upper Limb Venous Thrombosis in Paget-Schroetter Syndrome

**DOI:** 10.7759/cureus.13187

**Published:** 2021-02-07

**Authors:** Olusola Adekoya

**Affiliations:** 1 Internal Medicine, Kettering Medical Center, Dayton, USA

**Keywords:** paget-schroetter syndrome, thrombosis, thrombolysis, deep vein thrombosis, dvt

## Abstract

Deep venous thrombosis (DVT) of the upper extremities is usually secondary to inflammatory processes, malignancy, immobility from trauma, and inherited or acquired thrombophilias. This is a case of a young man who presented to our facility complaining of upper extremity pain and swelling. Imaging results showed thrombosis in the deep venous system of the left upper extremity, consistent with Paget-Schroetter syndrome.

## Introduction

Paget-Schroetter syndrome is an acute, effort-induced thrombosis of the deep veins of the upper extremity, occurring after intense physical use such as sporting activities [1]. It makes up 1%-4% of all cases of deep venous thrombosis (DVT) [2]. Usually, there is an anatomical factor which contributes to the etiology and progression of this syndrome, such as, a cervical rib or hypertrophied cervical and upper shoulder muscles. This begins a cascade of endothelial injury with eventual thrombus formation [1]. Upper extremity DVT especially in younger patients should raise suspicion of this syndrome.

## Case presentation

A 24-year-old healthy male with no medical history presented to the ED complaining of acute-onset, moderate pain in his left upper extremity, involving his shoulder. This began while he was lifting weights at the gym. He also complained of dependent edema and cyanotic discoloration which improved with elevation of the extremity. He denied any symptoms in the right upper extremity, neck, chest, or head.

His vital signs were stable at presentation. On physical examination, left upper extremity appeared cyanotic with 1+ non-pitting edema. Pulses were intact (2+) and equal on palpation of both radial and brachial arteries. Cyanosis improved with elevation of the left arm. Laboratory findings were within normal limits including a complete blood count and comprehensive metabolic panel. He had a slightly prolonged prothrombin time of 14.4 s (reference range 10.3--12.9 s), and an INR of 1.2 (reference range 0.9-1.1).

A CT-angiogram of his left upper extremity showed no abnormalities, and a venous ultrasound demonstrated extensive acute occlusive DVT of the left subclavian, axillary and brachial veins.

**Figure 1:**
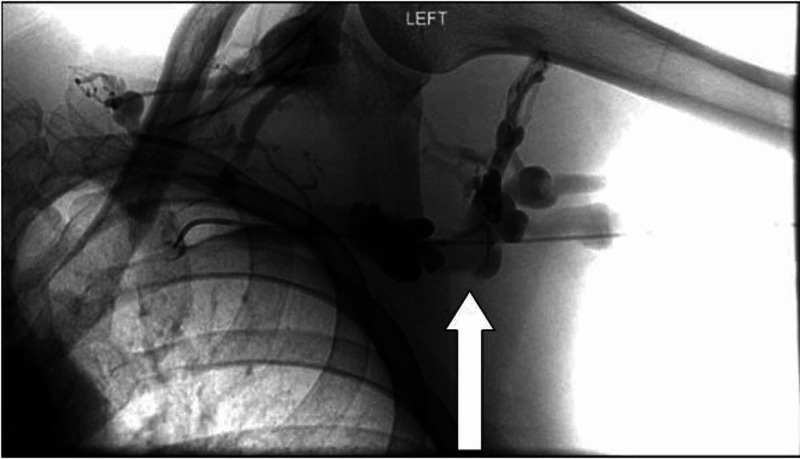
Left upper extremity venogram. Arrow showing thrombosis in the deep venous system before intervention.

He received systemic anticoagulation with heparin infusion, and also underwent catheter-directed thrombolysis after venography confirmed presence of DVT, as can be seen in Figure 1. 

**Figure 2:**
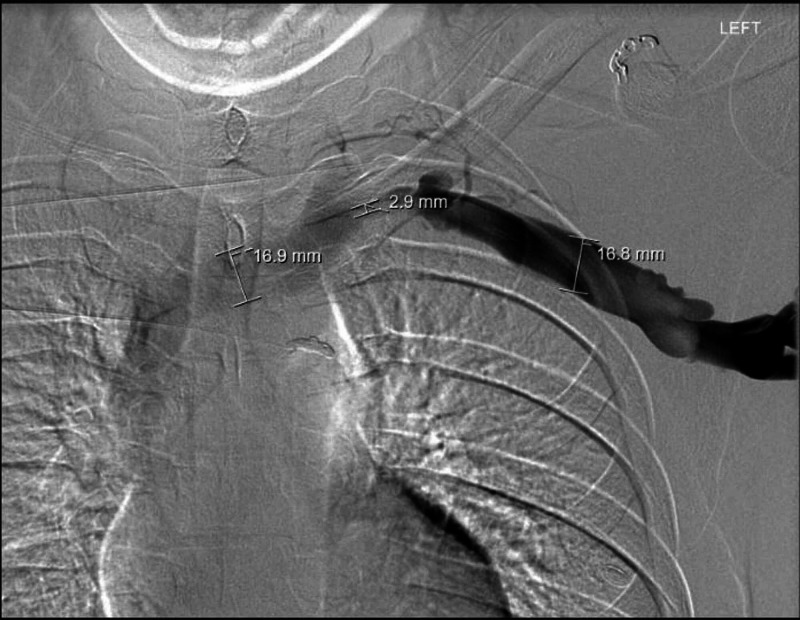
Post-thrombolysis venogram. Observed flow through the vein, and provided dimensions show stenosis at the area of the sternoclavicular junction.

After thrombolysis, venogram showed good results without residual venous thrombosis in the left upper extremity and central veins. However, severe stenosis of the left brachiocephalic vein at the level of the sternoclavicular junction was present. This is seen in Figure 2. 

The patient agreed to undergo first rib resection for definitive treatment, which was scheduled for a later date. He was transitioned to rivaroxaban, with plans to follow up for scheduled outpatient surgical management. Prior to discharge, the pain, swelling, and discoloration had improved. Investigation of thrombophilia was subsequently negative. 

## Discussion

Paget-Schroetter syndrome typically occurs in young and healthy patients who are physically active [1]. It makes up to 1%-4% of all DVTs and repetitive activity causing significant muscle hypertrophy and thoracic outlet obstruction, or other anatomical obstructions are usually associated risk factors [2]. 

Patients will usually present with complaints of post-workout pain, swelling, heaviness, and upper extremity discoloration [3]. Often, patients are able to relate onset of symptoms to physical activity. On inspection, these signs are apparent and it is extremely important to rule out arterial disease by physical examination and imaging studies, if uncertain. This will help prevent complications of arterial disease that could eventually lead to loss of limb when delayed.

Imaging modalities such as ultrasonography, catheter-directed venography, CT venography, and MR angiography are useful in making the diagnosis [4]. Treatment involves symptomatic management such as elevation of the extremity and pain management. Thrombolytic therapy followed by thoracic outlet decompression such as first rib resection is definitive management [5]. Prompt diagnosis and management are essential to avoid fibrosis and permanent disability [6].

## Conclusions

This case describes a typical presentation of Paget-Schroetter syndrome, highlights diagnosis, clinical course, and management in a young, otherwise healthy patient. It is vital to consider this diagnosis in patients presenting with upper extremity symptoms who are clinically consistent with DVT.

## References

[1] Alla VM, Natarajan N, Kaushik M, Warrier R, Nair CK (2010). Paget-Schroetter syndrome: review of pathogenesis and treatment of effort thrombosis. West J Emerg Med.

[2] Kommareddy A, Zaroukian MH, Hassouna HI (2002). Upper extremity deep venous thrombosis. Semin Thromb Hemost.

[3] Mall NA, Van Thiel GS, Heard WM, Paletta GA, Bush-Joseph C, Bach BR Jr (2013). Paget-Schroetter syndrome: a review of effort thrombosis of the upper extremity from a sports medicine perspective. Sports Health.

[4] Hangge P, Rotellini-Coltvet L, Deipolyi AR, Albadawi H, Oklu R (2017). Paget-Schroetter syndrome: treatment of venous thrombosis and outcomes. Cardiovasc Diagn Ther.

[5] Urschel HC, Jr Jr, Patel AN (2008). Surgery remains the most effective treatment for Paget-Schroetter syndrome: 50 years‘ experience. Ann Thorac Surg.

[6] Molina JE, Hunter DW, Dietz CA (2009). Protocols for Paget-Schroetter syndrome and late treatment of chronic subclavian vein obstruction. Ann Thorac Surg.

